# Research of Pathogenesis and Novel Therapeutics in Arthritis

**DOI:** 10.3390/ijms20071646

**Published:** 2019-04-02

**Authors:** Chih-Hsin Tang

**Affiliations:** 1 Department of Pharmacology, School of Medicine, China Medical University, Taichung 40402, Taiwan; chtang@mail.cmu.edu.tw; Tel.: +886-22052121 (ext. 7726); Fax: +886-4-22333641; 2 Chinese Medicine Research Center, China Medical University, Taichung 40402, Taiwan; 3 Department of Biotechnology, College of Health Science, Asia University, Taichung 41354, Taiwan

**Keywords:** rheumatoid arthritis, osteoarthritis, anti-arthritis, biomarkers

## Abstract

Arthritis has a high prevalence globally and includes over 100 types, the most common of which are rheumatoid arthritis, osteoarthritis, psoriatic arthritis and inflammatory arthritis. The exact etiology of arthritis remains unclear and no cure exists. Anti-inflammatory drugs are commonly used in the treatment of arthritis, but are associated with significant side effects. Novel modes of therapy and additional prognostic biomarkers are urgently needed for these patients. In this editorial, the twenty articles published in the Special Issue Research of Pathogenesis and Novel Therapeutics in Arthritis 2019 are summarized and discussed as part of the global picture of the current understanding of arthritis.

Arthritis has a high prevalence globally and includes over 100 types, the most common of which are rheumatoid arthritis (RA), osteoarthritis (OA), psoriatic arthritis and inflammatory arthritis. All types of arthritis share common features of disease, including monocyte infiltration, inflammation, synovial swelling, pannus formation, stiffness in the joints and articular cartilage destruction. The exact etiology of arthritis remains unclear and no cure exists. Anti-inflammatory drugs are commonly used in the treatment of arthritis, but are associated with significant side effects, such as gastric bleeding, an increased risk for heart attacks and other cardiovascular problems. Novel modes of therapy and additional prognostic biomarkers are urgently needed for these patients.

In response to the call for papers, we received many submissions from all over the world. After an initial screening, we selected 20 articles that are appropriate for this Special Issue. All manuscripts underwent a very rigorous peer-review process. The papers included in this issue can be broadly organized into three main categories: (i) the pathogenesis of arthritis, (ii) new biomarkers and (iii) novel strategies in the treatment of arthritis.

(i) Pathogenesis of arthritis. The important role of angiogenesis in arthritis progression has been summarized by MacDonald et al. [1]. The same research team has also summarized the critical role played by adipokines in cartilage and bone homeostasis in the pathogenesis of RA and OA, which has important implications for obesity [2]. The involvement of growth factors, inflammatory cytokines and differential miRNA expression in synovial tissue, articular cartilage and subchondral bone during the onset and progression of OA has been summarized by two research groups [3,4], while another research team has reviewed how the Epstein–Barr virus (EBV) is able to induce the onset of RA in predisposed shared epitope (SE)-positive individuals, by promoting entry of B-cells through direct contact between SE and gp42 in the entry complex [5]. An interesting article from Polish researchers reviews the evidence on the role of mesenchymal stromal cells in the pathogenesis of spondyloarthropathies (SpA) and discusses the potential use of stem cells in regenerative processes and the treatment of inflammatory changes in articular structures [6].

(ii) New biomarkers. Dudics et al. examined the miRNA expression profiles of immune cells from arthritic Lewis rats and arthritic rats treated with celastrol, a natural triterpenoid [7]. Their results indicate that several miRNAs may serve as novel biomarkers of disease activity and therapeutic response in autoimmune arthritis. Another article, by Chen et al., has explored the differential expression of novel miRNAs in RA osteoblasts [8]. The findings suggest that certain candidate genes may help in the evaluation of therapies targeting chemotaxis and neovascularization in an effort to control joint destruction in RA.

(iii) Novel strategies in the treatment of arthritis. Liu et al. describe the synthetization of an analogue, 6-(2,4-difluorophenyl)-3-(3-(trifluoromethyl)phenyl)-2H-benzo[e][1,3]oxazine-2,4(3H)-dione (Cf-02), which shares structural similarity with quercetin, a potent anti-inflammatory flavonoid present in many different fruits and vegetables [9]. Cf-02 was shown to suppress inflammation and cartilage damage. The methodology used by this research team shows considerable promise for the identification of candidate disease-modifying immunomodulatory drugs and lead compounds for arthritis therapies.

Tsai et al. have found that a natural diterpene compound, sclareol, inhibits the release of inflammatory cytokines (TNF-α and IL-6) in synovial fibroblasts and alleviates the severity of arthritis in an experimental model of RA, collagen-induced arthritis (CIA) [10], while the article by Jung et al. indicates that the active component of the herb *Dictamnus dasycarpus*, fraxinellone, alleviates synovial inflammation and osteoclastogenesis in CIA mice [11]. As for OA, Valenti et al. suggest that the bisphosphonate clodronate, already used in the treatment of osteoporosis, may prove to be a good therapeutic tool against OA [12].

Investigations by Wu et al. into the relationship between visfatin (a proinflammatory adipokine) and the expression of IL-6 and TNF-α describe how visfatin promotes their production in human synovial fibroblasts [13]. Another paper provides insight into the mechanism of crosstalk between IL-1β and WNT signaling in primary human chondrocytes, describing the pivotal roles played by inducible nitric oxide synthase (iNOS) and NO in in OA pathogenesis [14]. The evidence from these papers suggests that visfatin and iNOS/NO are novel therapeutic targets in arthritis.

Talotta et al. evaluated changes in percentages of T helper 9 (Th9) cells in response to an in vitro simulation assay that examined the immunogenicity of the infliximab originator (Remicade^®^) and its biosimilar compound (Remsima^®^), using peripheral blood mononuclear cells from a cohort of RA patients classified as infliximab responders or inadequate responders [15]. Their findings provide insights into the association between levels of Th9 cells, clinicopathological features of the patient cohort, their use of concomitant methotrexate and steroidal drugs, and the outcome of infliximab therapy.

Chen et al. summarize the current understanding of the immunopathogenic mechanisms underlying RA disease, which has led to the emergence of increasingly novel biologic agents for the treatment of RA [16]. Another article discusses the structural biology of TNF-α antagonists, including etanercept (Enbrel^®^), infliximab (Remicade^®^), adalimumab (Humira^®^), certolizumab-pegol (Cimzia^®^) and golimumab (Simponi^®^), all of which are used in the treatment of RA [17]. A review by Nandakumar suggests that it is worthwhile targeting pathogenic IgG molecules in arthritis through the process of glyco-engineering, using bacterial enzymes to specifically cleave IgG/alter N-linked Fc-glycans at Asn 297, or by blocking the downstream effector pathways; these techniques offer new avenues for developing novel therapeutics for arthritis treatment [18]. On this theme, one of the articles in this Special Issue details the potent pharmacodynamic effects, toxicity, and clinical translation of triptolide, a major extract of the herb *Tripterygium wilfordii* Hook F (TWHF), in RA treatment [19]. Makino et al. look to the future with their review of the evidence on novel biological enhancement strategies for spinal degenerative disease [20].

We hope that this collection of research will provide new impetus and directions for all those who are interested in the development of novel prevention and treatment strategies for arthritis.
